# Latest Insights on the Etiology and Management of Primary Adrenal Insufficiency in Children

**DOI:** 10.4274/jcrpe.2017.S002

**Published:** 2017-12-30

**Authors:** Tülay Güran

**Affiliations:** 1 Marmara University Faculty of Medicine, Department of Pediatric Endocrinology and Diabetes, İstanbul, Turkey

**Keywords:** Primary adrenal insufficiency, children, etiology, treatment

## Abstract

Primary adrenal insufficiency (PAI) is a heterogeneous group of disorders characterized by an impaired production of cortisol and other steroid hormones by the adrenal cortex. Most of the causes of PAI in childhood are inherited and monogenic in origin and are associated with significant morbidity and mortality whenever the diagnosis and treatment is delayed. Therefore, early and accurate diagnosis would allow appropriate management for the patients and genetic counselling for the family. Congenital adrenal hyperplasia accounts for most cases of PAI in childhood, followed by abnormalities in the development of the adrenal gland, resistance to adrenocorticotropin hormone action and adrenal destruction. In recent years, the use of genome-wide, next-generation sequencing approaches opened new avenues for identifying novel genetic causes in the PAI spectrum. Understanding the genetic basis of adrenal disorders is key to develop innovative therapies for patients with PAI. The promising progress made in congenital adrenal hyperplasia treatment brings new perspectives for personalized treatment in children with PAI. The aim of this review is to characterize recent advances in the genetics and management of PAI in children.

## INTRODUCTION

Primary adrenal insufficiency (PAI) is a relatively rare but potentially lethal clinical condition in which the adrenal cortex cannot produce adequate amounts of steroid hormones, primarily cortisol, but may also include impaired production of aldosterone and adrenal sex steroids. Recent molecular advances have expanded our knowledge of the etiologies of PAI. However, its diagnosis may be missed or delayed unless an illness or stress precipitates a severe cardiovascular collapse resulting in acute adrenal crisis. Early recognition of the clinical findings and treatment with glucocorticoids and rehydration with intravenous fluids, with or without mineralocorticoids and salt, are life-saving while attempts to confirm the diagnosis with extensive work-up are ongoing. Delay in treatment may result in disastrous clinical outcomes.

This review mainly focuses on the recent advances in the etiology, clinical manifestations and management of PAI of genetic origin in children.

### Etiology

PAI in children may arise from abnormalities in the development of adrenal gland, impaired steroidogenesis, resistance to adrenocorticotropin hormone (ACTH) action [familial glucocorticoid deficiency (FGD)] or adrenal destruction. In contrast to the predominance of autoimmune etiologies in adults, most causes of PAI in childhood are inherited and monogenic in origin (1,2,3,4). Table 1 summarises the aetiologies of inherited PAI in children.

### Congenital Adrenal Hyperplasia

**Figure 1:**
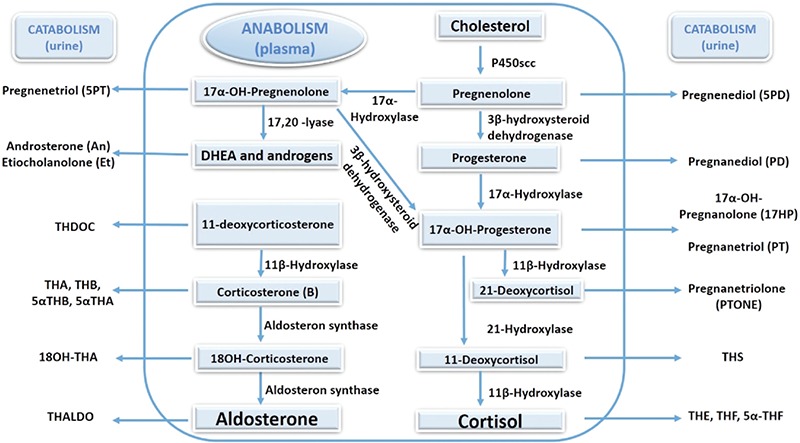
Adrenal steroidogenesis showing the main enzymatic steps of the pathway, intermediate precursors measurable in plasma and their respective catabolic metabolites detectable in the urine P450scc: P450 side chain cleavage, DHEA: dehydroepiandrosterone, THDOC: tetrahydrodeoxycorticosterone, 18OH-THA: 18OH-tetrahydrodehydrocorticosterone, (5α)THA: (5α)tetrahydrodehydrocorticosterone, (5β)THB: (5β)tetrahydrocorticosterone, THALDO: tetrahydroaldosterone, THS: tetrahydrodeoxycortisol, THE: tetrahydrocortisone, (5α)THF: (5α)tetrahydrocortisol

Congenital adrenal hyperplasia (CAH), which occurs in 1 in 10.000-18.000 live births, accounts for most cases of PAI in childhood (5). CAH represents a group of autosomal recessive disorders associated with deficiencies in the enzymes and cofactor proteins required for cortisol biosynthesis (6). Cortisol deficiency increases ACTH production that subsequently leads to adrenocortical hyperplasia and accumulation of the upstream precursor steroids above the enzyme deficiency. The accumulated upstream steroids and their urinary metabolites present the biochemical fingerprints for the localization of the defect (Figure 1). Additionally, these steroid precursors are generally diverted to androgen producing alternate pathways leading to androgen excess. The accumulation of certain steroid precursors enable differentiation of steroidogenic enzyme deficiencies (except StAR and P450 side-chain cleavage enzyme deficiencies) from the rest of the etiologies leading to PAI, as non-CAH is characterized by elevated ACTH concentrations and low steroidogenic intermediates.

The presence of hyperpigmentation of skin, nail beds, mucous membranes, palmar creases and scars is one of the hallmarks of primary adrenocortical pathology. ACTH and alpha-melanocyte stimulating hormone (α-MSH) are cleavage products of pro-opiomelanocortin (POMC). In patients with low cortisol levels as a consequence of adrenal disorders, POMC synthesis and consequently ACTH and MSH levels rise by negative feedback mechanisms. α-MSH then binds to the melanocortin 1 receptor on melanocyte cells, inducing a switch from the production of the pale skin pigment pheomelanin to eumelanin which is the darker (brown or black) pigment (7).

Clinical presentation may be mild or severe depending on the degree of impairment of enzyme activity and there may be signs, symptoms and laboratory findings of cortisol deficiency, mineralocorticoid deficiency or excess, undervirilization or androgen excess in males and sexual infantilism or virilization in affected females. The main signs and symptoms of cortisol deficiency include anorexia, weight loss, fatigue, myalgia, joint pain, low blood pressure, orthostatic hypotension, hyponatremia, hypoglycemia, lymphocytosis and eosinophilia and in addition direct hyperbilirubinemia and apnea may be present in newborn babies. Mineralocorticoid synthesis and release is under the control of the renin-angiotensin system, rather than ACTH. Therefore, mineralocorticoid deficiency develops only in adrenocortical abnormalities. Mineralocorticoid deficiency causes failure to thrive, abdominal pain, nausea, vomiting, dizziness, low blood pressure, orthostatic hypotension, hyponatremia, salt craving, hyperkalemia, metabolic acidosis, dehydration and hypovolemic shock. Lack of pubic and/or axillary hair and absent/delayed clinical adrenarche in either sex suggests deficiency of adrenal sex steroids.

More than 95% of all cases of CAH are caused by 21-hydroxylase deficiency (21-OHD). 21-OHD is classified into 3 subtypes according to retained enzyme activity and clinical severity: classic salt wasting, classic simple virilizing, and nonclassic CAH (NCCAH; mild or late onset) (6,8). The classic type affects approximately 1 in 16.000 live births. NCCAH is one of the most common autosomal recessive disorders in humans and affects approximately 1 in 1000 individuals (6). The second most common form of CAH, 11β-hydroxylase deficiency (11-OHD), occurs in 1 in 100,000 live births and accounts for approximately 5% of cases (9). Other less common forms of CAH include 3β-hydroxysteroid dehydrogenase type 2 deficiency, 17α-hydroxylase deficiency, POR deficiency, lipoid CAH and cholesterol side-chain cleavage enzyme deficiency. Distinctive clinical and biochemical features and management goals of CAH are presented in Table 2. An expert review on the genetic features of CAH is also available (6).

Advances in steroid assays in recent years, particularly the clinical utility of liquid chromatography/tandem mass spectrometry (LC-MS/MS), have allowed more accurate quantitation of key steroids, simultaneous measurement of multiple steroids from small biological samples and identification of novel steroids in the pathogenesis of adrenal disorders. The best example of this is the emerging evidence of 11-oxygenated 19-carbon (11oxC19) adrenal-derived steroids as clinically important androgens. 11oxC19 steroids are synthesized by the action of cytochrome P450 11β-hydroxylase. Besides the last step of cortisol biosynthesis, cytochrome P450 11β-hydroxylase mediates the conversion of androstenedione and testosterone into their respective 11-oxygenated products, namely 11β-hydroxyandrostenedione (11OHA4) and 11β-hydroxytestosterone (11OHT). These steroids are further converted to small amounts of 11-ketoandrostenedione (11KA4) and 11-ketotestosterone (11KT) respectively, by the action of 11β-hydroxysteroid dehydrogenase, type 2. 11oxC19 steroids are produced almost exclusively from the adrenal gland and they were shown to be three to four times higher in 21OHD patients than in controls. In addition 11KT was found to be more closely associated with poor control in 21OHD than testosterone levels in both males and females. Therefore it has been hypothesized that 11KT is a major adrenal androgen, responsible for suppression of gonadal functions observed in poorly controlled 21OHD (10). Furthermore, 21-deoxycortisol and 11oxC19 steroids showed the closest correlation with adrenal gland size and 11oxC19 steroids were detected at much higher concentrations in CAH patients with testicular adrenal rest tumor (TART) than those without (11). These findings suggest that 11oxC19 steroids may present clinically promising biomarkers in the treatment monitoring and management of CAH.

### Adrenal Dysgenesis/Hypoplasia

During the last two decades, high-throughput sequencing approaches proved very effective in reaching a molecular diagnosis for several forms of primary adrenal hypoplasia and adrenal dysgenesis syndromes. The genetic basis for these disorders involves various cellular and physiologic processes, including metabolism, nuclear protein import, oxidative stress defense mechanisms and regulation of cell cycle (12). Two distinct histological patterns of adrenal hypoplasia have been described; the miniature adult and cytomegalic forms. In the miniature adult form, adrenal cortex has normal structural organization whereas in the cytomegalic form of primary adrenal hypoplasia the residual adrenal cortex is structurally disorganized with scattered irregular nodular formations of eosinophilic cells, with the adult permanent zone absent or nearly absent. The miniature adult form is generally sporadic or inherited in an autosomal recessive manner while the cytomegalic form is generally considered to be X-linked, but there may be one or more autosomal genes associated with this phenotype (12). Regardless of underlying genetic etiology, conditions with adrenal hypoplasia/dysplasia are associated with deficiency of all adrenocortical hormones (aldosterone, cortisol, androgens). Most common is DAX1 deficiency which is due to genetic defects in NR0B1, located on chromosome Xp21.2. DAX1 defects have been detected in two thirds of males with PAI of unknown etiology by clinical or biochemical phenotype (13). Therefore all male patients with a history of non-CAH PAI should be screened for DAX1 deficiency, especially those with infertility, delayed/absent puberty or adrenal insufficiency in males from the maternal family. Adrenal insufficiency shows a bimodal distribution pattern of age at presentation ie either around newborn period or after 1 year of age. However late-onset DAX1 deficiency cases are also being increasingly reported from adult clinics (3,14). Patients with DAX1 deficiency present with variable phenotypes. Typically, they develop severe primary adrenal failure with salt-wasting. The hypogonadotropic hypogonadism may manifest as delayed puberty, impaired spermatogenesis or infertility which is explained by the expression of NR0B1 in the hypothalamus and the anterior pituitary, besides the adrenal glands and the gonads. Therefore, long-term focus on puberty and fertility is needed in affected individuals. Ambiguous genitalia is not a feature of DAX1 deficiency. However micropenis and or cryptorchidism may be present. Patients with precocious puberty have also been reported (15,16). Although this is an X-linked condition, females carrying homozygous or heterozygous mutations have also been reported to express phenotypic features of adrenal hypoplasia congenital due to non-random X inactivation (17,18). Genetic counselling can help to identify family members at risk of adrenal insufficiency and female carriers.

The SF1 protein, encoded by the nuclear receptor subfamily 5, group A, member 1 (NR5A1) gene, is expressed in the adrenal gland, gonads, hypothalamus and anterior pituitary. SF1 has a crucial role in adrenal gland, gonads and spleen development in both sexes. Besides, SF1 is involved in the regulation of energy balance and glucose homeostasis in the central nervous system (19). SF1 deficiency develops as a result of pathogenic mutations in NR5A1 gene in both heterozygous or homozygous inheritance. In contrast to DAX1-associated diseases, SF-1 deficiency only rarely causes adrenal insufficiency, but generally in combination with testicular dysgenesis. Isolated adrenal failure has rarely been reported (20). However, long-term follow-up for adrenal function is important for those patients with NR5A1 mutations. Phenotypic features in 46,XY individuals with NR5A1 mutations include different forms of disorders of sex differentiation (DSD) ranging from hypospadias to complete female phenotype or late-onset impaired spermatogenesis and infertility. NR5A1 gene defects should also be considered in 46,XY DSD cases with normal testosterone concentrations, similar to androgen receptor (AR) mutations or mild 5-α reductase, or mild 17-ketosteroid reductase deficiencies. Mutations in NR5A1 were found in 46,XX females with isolated/premature ovarian insufficiency (14). 46,XX testicular/ovotesticular DSD is also described in one case (21,22). Poly/asplenia can be seen in both sexes (23).

The common feature of syndromes associated with adrenal hypoplasia is the severe impairment of growth and tissue development and particularly with a prenatal onset. These disorders specifically impair the machinery involved in cell division and cell cycling. The author suggests evaluation of adrenal function in any patient with severe, prenatal-onset growth retardation and with syndromic features, especially with cerebral and finger malformations (Table 1).

Here, two specific examples of syndromic adrenal hypoplasia are given.

IMAGe syndrome is a recently described, syndromic adrenal hypoplasia syndrome associated with severe growth failure. This syndrome develops as a result of impaired expression of a cell cycle regulator protein, cyclin dependent kinase inhibitor 1C (CDKN1C). CDKN1C, encoded by the CDKN1C gene, is a negative regulator of cell proliferation maintaining the cell at the non-proliferative state throughout life. The loss-of-function mutations, located at the CDK-binding domain of the CDKN1C gene, are associated with Beckwith-Wiedemann syndrome. Recently, gain-of-function mutations in the PCNA domain of CDKN1C have been have been described in association with various growth-retarded syndromes including IMAGe syndrome and Russell Silver syndrome as well as a novel undergrowth syndrome that additionally exhibits early adulthood onset diabetes (24). De novo heterozygous CDKN1C mutations or imprinted mode of inheritance with maternal transmission of CDKN1C mutations were reported. Early recognition of metaphyseal dysplasia accompanying early-onset, severe adrenal insufficiency is crucial for the diagnosis IMAGe syndrome. Delayed endochondral ossification, osteopenia, hypercalcemia, and/or hypercalciuria of variable degree are among the early findings. Dysmorphic craniofacial features including prominent forehead, low-set ears, short nose, flat nasal bridge, rhizomelic shortening and genital abnormalities in males are other associated features.

Another severe growth-restricting pathology associated with adrenal hypoplasia has recently been described in patients due to gain-of-function mutations in the SAMD9 gene. Growth and survival is so impaired in this genetic disorder that affected individuals develop tissue adaptation by progressive loss of mutated SAMD9 in chromosomal structure. This modification is achieved through the development of monosomy 7 (-7), deletions of 7q (7q-), and secondary somatic loss-of-function (nonsense and frameshift) mutations in SAMD9 to rescue the growth-restricting effects of mutant SAMD9 proteins in bone marrow and to increase the length of survival (25). So the use of advanced diagnostic and molecular technologies has helped to define novel mechanisms in human development beyond genetic defects in adrenal development and adrenal steroidogenesis.

Affected individuals with heterozygous gain-of-function mutation in SAMD9 present with MIRAGE syndrome, which is an acronym of myelodysplasia, infection, restriction of growth, adrenal hypoplasia, genital phenotypes, and enteropathy (26).

### Adrenocorticotropin Hormone Resistance

Mutations in MC2R (encoding the ACTH receptor protein, MC2R) and MRAP (encoding MC2R accessory protein) are well described causes of inherited disorders of ACTH binding and signaling, namely FGD type 1 (FGD1) and type 2 (FGD2). FGD is characterized by cortisol deficiency together with a preserved renin-aldosterone axis. Children typically present with hypoglycemia or hyperpigmentation in early infancy or in childhood. Some associated phenotypical features may also be present (Table 1). Children with FGD do not typically have salt-loss. However, transient hyponatremia has been reported in several children with severe MC2R defects, sometimes leading to a misdiagnosis of adrenal hypoplasia (3). Plasma ACTH often remains markedly raised despite normal or even supranormal glucocorticoid treatment. Therefore, affected patients remain hyperpigmented. So the clinical aim of glucocorticoid replacement strategies should not be to suppress ACTH or normalization of hyperpigmentation but should rather target normal water and electrolyte balance and a normal physical growth rate.

### Mitochondria and Adrenal Gland

**Figure 2:**
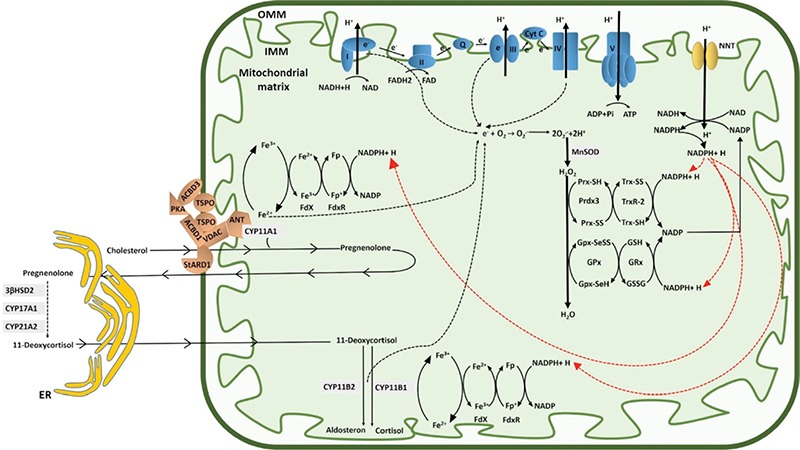
Mitochondrial machinery involved in the regulation of steroidogenesis. Access of cholesterol to the mitochondria is regulated by the steroidogenic acute regulatory protein, (StAR), serving as the acute regulator of steroidogenesis. StAR action requires interaction with the transduceosome complex that is composed of a group of proteins (translocator protein, voltage dependent anion channel 1, ACBD3, adenine nucleotide transporter, protein kinase A) at the inner mitochondrial membrane in the process of transporting cholesterol molecules directly to the cholesterol side chain cleavage enzyme, P450scc (CYP11A1) to initiate steroidogenesis. CYP11A1 is the enzymatic rate-limiting step in steroidogenesis which determines cellular steroidogenic capacity. CYP11A1, CYP11B1 and CYP11B2 are the main mitochondrial cytochrome P450 enzymes involved in steroidogenesis. Four complexes of the electron transport chain (indicated in blue) transfer electrons to generate energy required for various cellular processes including steroid biosynthesis. Nicotinamide nucleotide transhydrogenase (NNT), is an integral protein of the inner mitochondrial membrane. This enzyme uses energy from the mitochondrial proton gradient to produce high concentrations of nicotinamide adenine dinucleotide phosphate (NADPH). NADPH is the electron supplier for two electron-transfer intermediates, ferredoxin reductase and ferredoxin which are required for mitochondrial P450 enzymes to produce steroid hormones. NADPH is also used by mitochondrial antioxidant defence machinery, comprising glutathione peroxidase (Gpx) and the peroxiredoxin-thioredoxin systems, which are responsible for the inactivation of reactive oxygen species derived from the leakage of electrons from electron transport chain during energy generation procedures. Genetic defects in many components of this machinery (including StAR, CYP11A1, CYP11B1, NNT, TXNRD2, GPX1, PRDX3) have been described in patients with primary adrenal insufficiency TSPO: translocator protein, VDAC: voltage dependent anion channel, ANT: adenine nucleotide transporter, PKA: protein kinase A; FdXR: ferredoxin reductase, FdX: ferredoxin, Gpx: glutathione peroxidase, NADPH: nicotinamide adenine dinucleotide phosphate, NNT: Nicotinamide nucleotide transhydrogenase, StAR: steroidogenic acute regulatory protein, Prdx-Trx: peroxiredoxin-thioredoxin

Recent advances in molecular studies and application of genome-wide, next-generation sequencing approaches revealed the importance of mitochondrial function for endocrine health and steroid hormone biosynthesis. All steroid hormones are synthesized within mitochondria by tissue-specific steroidogenic enzymes (Figure 2). Mitochondrial dysfunction may affect the capacity for adrenocortical hormone production by impaired mitochondrial ATP production, oxidative stress and/or accelerated apoptosis (27). In particular some of the latest findings have expanded the spectrum of pathogenetic mechanisms causing adrenal disease and imply that the adrenal is highly vulnerable to oxidative stresses (Figure 2) (28,29).

Molecular defects in both mitochondrial and nuclear genomes have been associated with mitochondrial dysfunction (Table 1). Clinicians should have a high level of suspicion for the possibility of an underlying mitochondrial disease in patients with adrenal insufficiency associated with sensorineural hearing loss, lactic acidosis and accompanying endocrine abnormalities (diabetes, hypoparathyroidism, hypogonadism, hypothyroidism) and multisystemic diseases (epilepsy, stroke, encephalopathy, cranial abnormalities, cardiac conduction defects, neuropathy, retinopathy).

### Sphingolipids and Adrenal Gland

The essential role of sphingolipid metabolism has recently emerged in adrenal disease. Congenital sphingosine1phosphate (S1P) lyase deficiency due to biallelic mutations in the SGPL1 gene has been described, in association with PAI and steroid-resistant nephrotic syndrome (30,31,32). S1P lyase is the enzyme responsible for irreversible S1P degradation which is the final step in sphingolipid breakdown. Inhibition of S1P lyase activity will lead to accumulation of bioactive signaling molecules upstream of the pathway including S1P and ceramides (Cer). We have recently demonstrated that accumulation of S1P, Cer and potentially other upstream components of the sphingolipid pathway, due to congenital S1P lyase deficiency, leads to a multisystemic disorder including PAI, nephrotic syndrome and ichthyosis, primary hypothyroidism, cryptorchidism, lymphopenia and neurological anomalies.

**Figure 3:**
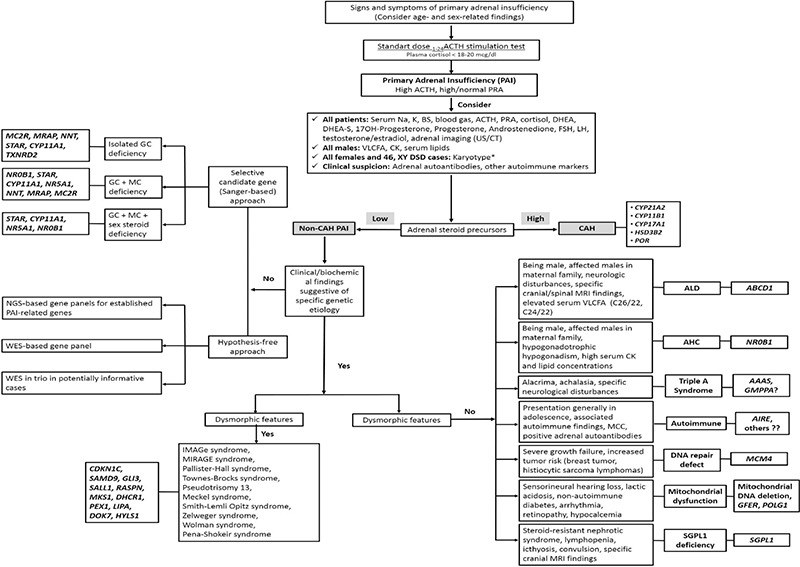
A proposed diagnostic work up algorithm for targetted genetic testing to determine the etiologic diagnosis in inherited primary adrenal failure in children *Karyotype can be excluded in female phenotype patients whenever pelvic US confirms the presence of normal ovaries and Mullerian structures. Assessment of karyotype-matched normal external and internal genitalia and gonads is crucial for deciding about gonadal sex steroid production ACTH: adrenocorticotropin, PRA: plasma renin activity, Na: sodium, K: potassium, BS: blood sugar, DHEA: dehydroepiandrosterone, FSH: follicle-stimulating hormone, LH: luteinizing hormone, VLCFA: very-long-chain fatty acids, CK: creatinine kinase, US: ultrasound, CT: computerized tomography, CAH: congenital adrenal hyperplasia, GC: glucocorticoid, MC: mineralocorticoid, MRI: magnetic resonance imaging, ALD: adrenoleukodystrophy, AHC: adrenal hypoplasia congenital, MCC: mucocutaneous candidiasis, NGS: next generation sequencing, WES: whole exome sequencing, SGPL1: sphingosine-1-phosphate lyase

Establishing a specific genetic diagnosis of PAI is extremely valuable for identifying presymptomatic children who could benefit from treatment before the onset of potentially life-threatening symptoms and for counseling family members appropriately about the risk of passing the condition on to their children. Knowing the genetic etiology can also help to modify treatments, such as the need for long-term mineralocorticoid replacement, and can predict potential co-morbidities, such as impaired puberty or fertility and neurological dysfunction. An etiological approach in children with Inherited Primary Adrenal Insufficiency is suggested in Figure 3.

### Treatment

Replacement of glucocorticoids and mineralocorticoids, particularly by hydrocortisone and fludrocortisone is the mainstay of treatment in adrenal insufficiency. Intravenous fluids and salt replacement should be added to the treatment in stressful conditions and adrenal crisis. Principal treatment goals include maintaining a physiologic water and electrolyte homeostasis together with attainment of normal physical and pubertal growth. CAH management should also target reduction of androgen exposure. Additionally, optimization of hydrocortisone treatment is critical to mimic the physiological circadian rhythm of cortisol secretion and to avoid excessive glucocorticoid exposure which is associated with poor long-term health outcomes, including growth suppression, obesity, metabolic syndrome, diabetes and osteoporosis (33). These challenges have led to the development of new glucocorticoid formulations and some adjuvant treatments (34). In recent years, investigators have developed two modified-release, oral, glucocorticoid preparations. The first is a dual-release hydrocortisone with an extended-release core surrounded by an immediate-release coating (Plenadren; ViroPharma, Maidenhead, UK), which was developed for once-daily, first-morning administration in patients with PAI. However, it is unable to deliver a sufficient early morning cortisol rise and to suppress ACTH and adrenal androgens in the morning by once-daily dosing. Plenadren failed to achieve physiologic cortisol replacement in a small case series of children with non-CAH primary adrenal failure and secondary adrenal insufficiency (35,36,37). Plenadren is not yet licensed for use in the management of adrenal insufficiency in children, but is available for use in adult patients with a good safety profile (38). The second formulation is a delayed and sustained release, multiparticulate hydrocortisone, Chronocort® (Diurnal, UK). Chronocort given at morning and night doses provides release of hydrocortisone in the early hours of the morning, replicating a physiological cortisol secretion pattern. It also appears to achieve better control of excessive androgen synthesis produced via classical and alternative pathways through attenuation of androstenedione and 17OH-progesteron (39). There is an ongoing phase III study to evaluate long-term effects of Chronocort treatment. This drug is also not licenced for use in children. There are a few recent trials to evaluate the bioavailability and absorption of modified hydrocortisone formulations, such as granules or sprinkles, for young children (Infacort®, Diurnal Ltd) (40). Continuous subcutaneous hydrocortisone infusion (CSHI) via a pump, similar to an insulin pump, is superior in achieving a better cortisol secretion profile and lowering ACTH concentrations in non-CAH PAI and in lowering serum androgens in CAH (41,42). However, certain issues limit the use of CSHI including high cost, complexity of device usage, the need for patient/parent education, the potential for local irritation and the potential for uninterrupted equipment wear and malfunction which would be particularly risky in patients with complete glucocorticoid deficiency. A recent meta-analysis demonstrated that extended-/dual-release and CSHI forms of glucocorticoid treatments are associated with higher life quality scores over the short-term (43).

Non-glucocorticoid adjuvant pharmacologic treatments for adrenal failure mainly target control of hyperandrogenism in CAH (34). Among them, abiraterone may be a promising alternative therapy that decreases the need for supraphysiologic exogenous glucocorticoids. Abiraretone is a potent inhibitor of CYP17A1, required for the synthesis of gonadal and adrenal androgens. Combined use of abiraterone with glucocorticoids can effectively lower androstenedione and testosterone metabolites in adult women with 21OHD without any potential side effects including hypertension and hypokalemia. However, it does not lower ACTH and inhibits gonadal sex-steroid secretion which limits its use in males with TART and for patients who desire fertility (44). A CRH receptor-1 antagonist was used in a Phase 1 trial of eight CAH women at a single dose which showed a 40% reduction in morning ACTH rise to control hyperandrogenism (45).

## CONCLUSION

PAI is a relatively rare but potentially lethal clinical condition in children. Early recognition of adrenal insufficiency can be difficult, although treatment is usually successful once it is initiated and, in most cases, lifelong treatment is necessary. Monogenic conditions, particularly CAH, account for most cases of PAI in childhood. Application of omics-based approaches by LC combined with MS significantly facilitated the recognition of biochemical markers of various steroidogenic enzyme deficiencies. In particular, targeted LC-MS/MS steroid panels, besides being very well suited for the routine laboratory setting, have proven extremely useful in diagnosing CAH subtypes and guiding treatment. However, non-CAH PAI often remains without a definite cause in a substantial number of cases. Detailed clinical phenotyping of such cases is critically important for diagnostic workflow but genotyping is equally important, confirming the diagnosis or carrier state, providing prognostic information on disease severity and is essential for genetic counseling.

Adrenal insufficiency is associated with a reduced quality of life that may be caused by non-physiological glucocorticoid replacement. In recent years, a substantial amount of progress has been made in optimizing glucocorticoid delivery systems, as well as by exploring non-glucocorticoid therapeutic strategies in CAH. However, there is still a long way to go in developing disease-specific and personalized treatments for children with PAI.

## Figures and Tables

**Table 1:**
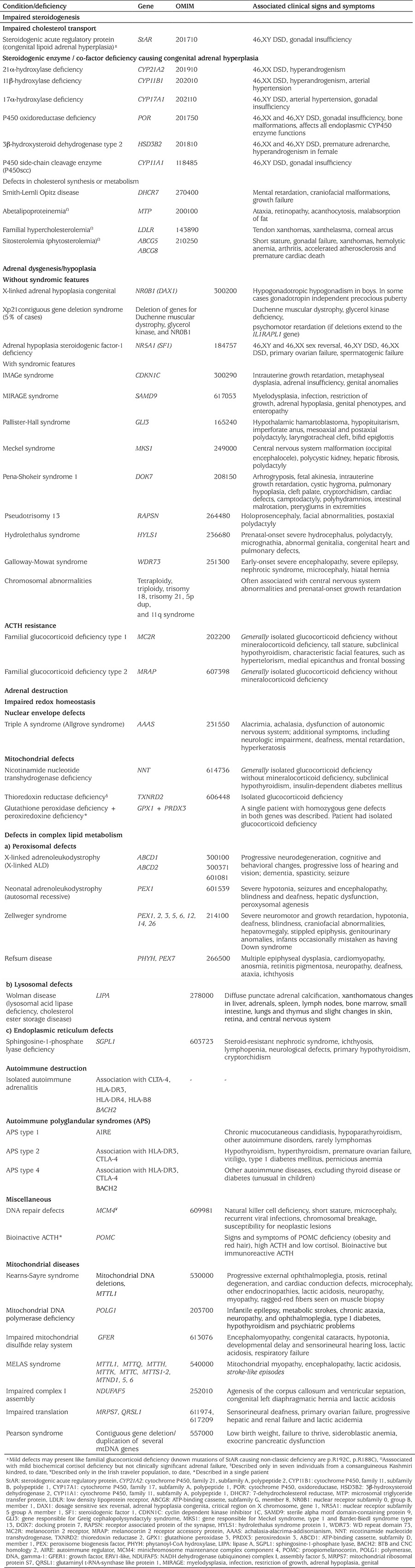
Aetiologies of inherited primary adrenal insufficiency in children

**Table 2:**
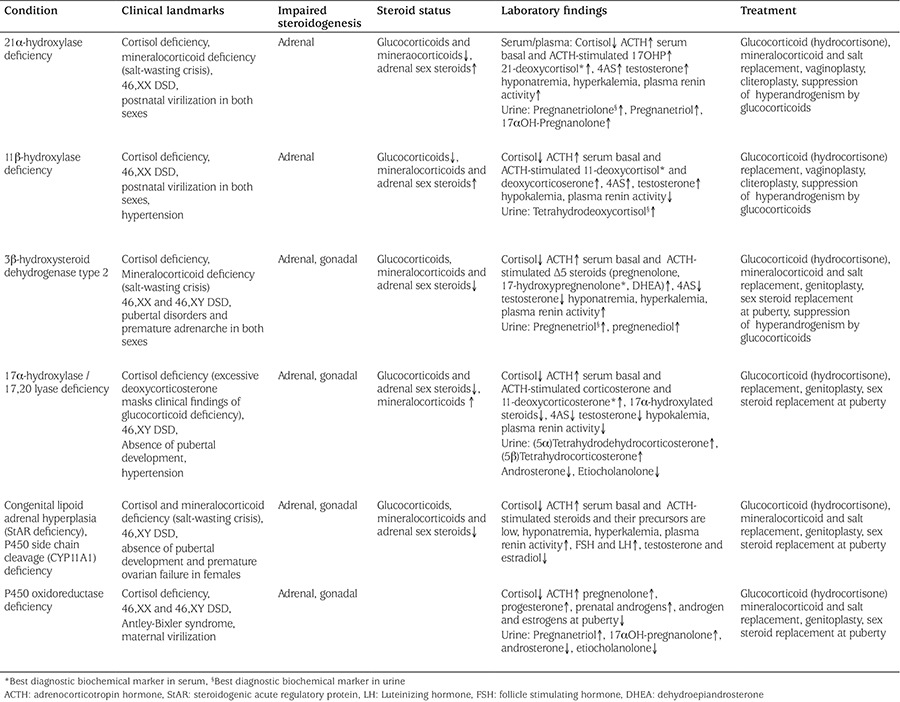
Clinical and laboratory findings of different forms of congenital adrenal hyperplasia and treatment goals

## References

[1] Perry R, Kecha O, Paquette J, Huot C, Van Vliet G, Deal C (2005). Primary adrenal insufficiency in children: twenty years experience at the Sainte-Justine Hospital, Montreal. J Clin Endocrinol Metab.

[2] Hsieh S, White PC (2011). Presentation of primary adrenal insufficiency in childhood. J Clin Endocrinol Metab.

[3] Guran T, Buonocore F, Saka N, Ozbek MN, Aycan Z, Bereket A, Bas F, Darcan S, Bideci A, Guven A, Demir K, Akinci A, Buyukinan M, Aydin BK, Turan S, Agladioglu SY, Atay Z, Abali ZY, Tarim O, Catli G, Yuksel B, Akcay T, Yildiz M, Ozen S, Doger E, Demirbilek H, Ucar A, Isik E, Ozhan B, Bolu S, Ozgen IT, Suntharalingham JP, Achermann JC (2016). Rare Causes of Primary Adrenal Insufficiency: Genetic and Clinical Characterization of a Large Nationwide Cohort. J Clin Endocrinol Metab.

[4] Amano N, Narumi S, Hayashi M, Takagi M, Imai K, Nakamura T, Hachiya R, Sasaki G, Homma K, Ishii T, Hasegawa T (2017). Genetic defects in pediatric-onset adrenal insufficiency in Japan. Eur J Endocrinol.

[5] White PC, Speiser PW (2000). Congenital adrenal hyperplasia due to 21-hydroxylase deficiency. Endocr Rev.

[6] Hannah-Shmouni F, Chen W, Merke DP (2017). Genetics of Congenital Adrenal Hyperplasia. Endocrinol Metab Clin North Am.

[7] Park J, Didi M, Blair J (2016). The diagnosis and treatment of adrenal insufficiency during childhood and adolescence. Arch Dis Child.

[8] El-Maouche D, Arlt W, Merke DP (2017). Congenital adrenal hyperplasia. Lancet.

[9] Khattab A, Haider S, Kumar A, Dhawan S, Alam D, Romero R, Burns J, Li D, Estatico J, Rahi S, Fatima S, Alzahrani A, Hafez M, Musa N, Razzghy Azar M, Khaloul N, Gribaa M, Saad A, Charfeddine IB, Bilharinho de Mendonça B, Belgorosky A, Dumic K, Dumic M, Aisenberg J, Kandemir N, Alikasifoglu A, Ozon A, Gonc N, Cheng T, Kuhnle-Krahl U, Cappa M, Holterhus PM, Nour MA, Pacaud D, Holtzman A, Li S, Zaidi M, Yuen T, New MI (2017). Clinical, genetic, and structural basis of congenital adrenal hyperplasia due to 11β-hydroxylase deficiency. Proc Natl Acad Sci USA.

[10] Turcu AF, Nanba AT, Chomic R, Upadhyay SK, Giordano TJ, Shields JJ, Merke DP, Rainey WE, Auchus RJ (2016). Adrenal-derived 11-oxygenated 19-carbon steroids are the dominant androgens in classic 21-hydroxylase deficiency. Eur J Endocrinol.

[11] Turcu AF, Mallappa A, Elman MS, Avila NA, Marko J, Rao H, Tsodikov A, Auchus RJ, Merke DP (2017). 11-Oxygenated Androgens Are Biomarkers of Adrenal Volume and Testicular Adrenal Rest Tumors in 21-Hydroxylase Deficiency. J Clin Endocrinol Metab.

[12] Kyritsi EM, Sertedaki A, Chrousos G, Charmandari E (2015). In: De Groot LJ, Chrousos G, Dungan K, Feingold KR, Grossman A, Hershman JM, Koch C, Korbonits M, McLachlan R, New M, Purnell J, Rebar R, Singer F, Vinik A, (eds). Familial Or Sporadic Adrenal Hypoplasia Syndrome. Endotext [Internet].

[13] Lin L, Gu WX, Ozisik G, To WS, Owen CJ, Jameson JL, Achermann JC (2006). Analysis of DAX1 (NR0B1) and steroidogenic factor-1 (NR5A1) in children and adults with primary adrenal failure: ten years’ experience. J Clin Endocrinol Metab.

[14] Suntharalingham JP, Buonocore F, Duncan AJ, Achermann JC (2015). DAX-1 (NR0B1) and steroidogenic factor-1 (SF-1, NR5A1) in human disease. Best Pract Res Clin Endocrinol Metab.

[15] Landau Z, Hanukoglu A, Sack J, Goldstein N, Weintrob N, Eliakim A, Gillis D, Sagi M, Shomrat R, Kosinovsky EB, Anikster Y (2010). Clinical and genetic heterogeneity of congenital adrenal hypoplasia due to NR0B1 gene mutations. Clin Endocrinol (Oxf).

[16] Durmaz E, Turkkahraman D, Berdeli A, Berdeli M, Karaguzel G, Akcurin S, Bircan I (2013). A novel DAX-1 mutation presented with precocious puberty and hypogonadotropic hypogonadism in different members of a large pedigree. J Pediatr Endocrinol Metab.

[17] Seminara SB, Achermann JC, Genel M, Jameson JL, Crowley WF Jr (1999). X-linked adrenal hypoplasia congenita: a mutation in DAX1 expands the phenotypic spectrum in males and females. J Clin Endocrinol Metab.

[18] Merke DP, Tajima T, Baron J, Cutler GB Jr (1999). Hypogonadotropic hypogonadism in a female caused by an X-linked recessive mutation in the DAX1 gene. N Engl J Med.

[19] Sohn JW, Oh Y, Kim KW, Lee S, Williams KW, Elmquist JK (2016). Leptin and insulin engage specific PI3K subunits in hypothalamic SF1 neurons. Mol Metab.

[20] El-Khairi R, Achermann JC (2012). Steroidogenic factor-1 and human disease. Semin Reprod Med.

[21] Bashamboo A, Donohoue PA, Vilain E, Rojo S, Calvel P, Seneviratne SN, Buonocore F, Barseghyan H, Bingham N, Rosenfeld JA, Mulukutla SN, Jain M, Burrage L, Dhar S, Balasubramanyam A, Lee B, Dumargne MC, Eozenou C, Suntharalingham JP, de Silva K, Lin L, Bignon-Topalovic J, Poulat F, Lagos CF, McElreavey K, Achermann JC, Members of UDN (2016). A recurrent p.Arg92Trp variant in steroidogenic factor-1 (NR5A1) can act as a molecular switch in human sex development. Hum Mol Genet.

[22] Baetens D, Stoop S, Peelman F, Todeschini AL, Rosseel T, Coppieters F, Veitia RA, Looijenga LH, De Baere E, Cools M (2017). NR5A1 is a novel disease gene for 46,XX testicular and ovotesticular disorders of sex development. Genet Med.

[23] Colson C, Aubry E, Cartigny M, Rémy AA, Franquet H, Leroy X, Kéchid G, Lefèvre C, Besson R, Cools M, Spinoit AF, Sultan C, Manouvrier S, Philibert P, Ghoumid J (2017). SF1 and spleen development: new heterozygous mutation, literature review and consequences for NR5A1-mutated patient’s management. Clin Genet.

[24] Cabrera-Salcedo C, Kumar P, Hwa V, Dauber A (2017). IMAGe and Related Undergrowth Syndromes: The Complex Spectrum of Gain-of-Function CDKN1C Mutations. Pediatr Endocrinol Rev.

[25] Buonocore F, Kühnen P, Suntharalingham JP, Del Valle I, Digweed M, Stachelscheid H, Khajavi N, Didi M, Brady AF, Blankenstein O, Procter AM, Dimitri P, Wales JKH, Ghirri P, Knöbl D, Strahm B, Erlacher M, Wlodarski MW, Chen W, Kokai GK, Anderson G, Morrogh D, Moulding DA, McKee SA, Niemeyer CM, Grüters A, Achermann JC (2017). Somatic mutations and progressive monosomy modify SAMD9-related phenotypes in humans. J Clin Invest.

[26] Narumi S, Amano N, Ishii T, Katsumata N, Muroya K, Adachi M, Toyoshima K, Tanaka Y, Fukuzawa R, Miyako K, Kinjo S, Ohga S, Ihara K, Inoue H, Kinjo T, Hara T, Kohno M, Yamada S, Urano H, Kitagawa Y, Tsugawa K, Higa A, Miyawaki M, Okutani T, Kizaki Z, Hamada H, Kihara M, Shiga K, Yamaguchi T, Kenmochi M, Kitajima H, Fukami M, Shimizu A, Kudoh J, Shibata S, Okano H, Miyake N, Matsumoto N, Hasegawa T (2016). SAMD9 mutations cause a novel multisystem disorder, MIRAGE syndrome, and are associated with loss of chromosome 7. Nat Genet.

[27] Chow J, Rahman J, Achermann JC, Dattani MT, Rahman S (2017). Mitochondrial disease and endocrine dysfunction. Nat Rev Endocrinol.

[28] Meimaridou E, Kowalczyk J, Guasti L, Hughes CR, Wagner F, Frommolt P, Nürnberg P, Mann NP, Banerjee R, Saka HN, Chapple JP, King PJ, Clark AJ, Metherell MA (2012). Mutations in NNT encoding nicotinamide nucleotide transhydrogenase cause familial glucocorticoid deficiency. Nat Genet.

[29] Prasad R, Chan LF, Hughes CR, Kaski JP, Kowalczyk JC, Savage MO, Peters CJ, Nathwani N, Clark AJ, Storr HL, Metherell LA (2014). Thioredoxin Reductase 2 (TXNRD2) mutation associated with familial glucocorticoid deficiency (FGD). J Clin Endocrinol Metab.

[30] Prasad R, Hadjidemetriou I, Maharaj A, Meimaridou E, Buonocore F, Saleem M, Hurcombe J, Bierzynska A, Barbagelata E, Bergadá I, Cassinelli H, Das U, Krone R, Hacihamdioglu B, Sari E, Yesilkaya E, Storr HL, Clemente M, Fernandez-Cancio M, Camats N, Ram N, Achermann JC, Van Veldhoven PP, Guasti L, Braslavsky D, Guran T, Metherell LA (2017). Sphingosine-1-phosphate lyase mutations cause primary adrenal insufficiency and steroid-resistant nephrotic syndrome. J Clin Invest.

[31] Lovric C, Goncalves S, Gee HY, Oskouian B, Srinivas H, Choi WI, Shril S, Ashraf S, Tan W, Rao J, Airik M, Schapiro D, Braun DA, Sadowski CE, Widmeier E, Jobst-Schwan T, Schmidt JM, Girik V, Capitani G, Suh JH, Lachaussée N, Arrondel C, Patat J, Gribouval O, Furlano M, Boyer O, Schmitt A, Vuiblet V, Hashmi S, Wilcken R, Bernier FP, Innes AM, Parboosingh JS, Lamont RE, Midgley JP, Wright N, Majewski J, Zenker M, Schaefer F, Kuss N, Greil J, Giese T, Schwarz K, Catheline V, Schanze D, Franke I, Sznajer Y, Truant AS, Adams B, Désir J, Biemann R, Pei Y, Ars E, Lloberas N, Madrid A, Dharnidharka VR, Connolly AM, Willing MC, Cooper MA, Lifton RP, Simons M, Riezman H, Antignac C, Saba JD, Hildebrandt F (2017). Mutations in sphingosine-1-phosphate lyase cause nephrosis with ichthyosis and adrenal insufficiency. J Clin Invest.

[32] Janecke AR, Xu R, Steichen-Gersdorf E, Waldegger S, Entenmann A, Giner T, Krainer I, Huber LA, Hess MW, Frishberg Y, Barash H, Tzur S, Schreyer-Shafir N, Sukenik-Halevy R, Zehavi T, Raas-Rothschild A, Mao C, Müller T (2017). Deficiency of the sphingosine-1-phosphate lyase SGPL1 is associated with congenital nephrotic syndrome and congenital adrenal calcifications. Hum Mutat.

[33] Porter J, Blair J, Ross RJ (2017). Is physiological glucocorticoid replacement important in children?. Arch Dis Child.

[34] Turcu AF, Auchus RJ (2016). Novel treatment strategies in congenital adrenal hyperplasia. Curr Opin Endocrinol Diabetes Obes.

[35] Johannsson G, Bergthorsdottir R, Nilsson AG, Lennernas H, Hedner T, Skrtic S (2009). Improving glucocorticoid replacement therapy using a novel modified-release hydrocortisone tablet: a pharmacokinetic study. Eur J Endocrinol.

[36] Johannsson G, Nilsson AG, Bergthorsdottir R, Burman P, Dahlqvist P, Ekman B, Engström BE, Olsson T, Ragnarsson O, Ryberg M, Wahlberg J, Biller BM, Monson JP, Stewart PM, Lennernäs H, Skrtic S (2012). Improved cortisol exposure-time profile and outcome in patients with adrenal insufficiency: a prospective randomized trial of a novel hydrocortisone dual-release formulation. J Clin Endocrinol Metab.

[37] Giordano R, Guaraldi F, Marinazzo E, Fumarola F, Rampino A, Berardelli R, Karamouzis I, Lucchiari M, Manetta T, Mengozzi G, Arvat E, Ghigo E (2016). Improvement of anthropometric and metabolic parameters, and quality of life following treatment with dual-release hydrocortisone in patients with Addison’s disease. Endocrine.

[38] Nilsson AG, Bergthorsdottir R, Burman P, Dahlqvist P, Ekman B, Engström BE, Ragnarsson O, Skrtic S, Wahlberg J, Achenbach H, Uddin S, Marelli C, Johannsson G (2017). Long-term safety of once-daily, dual-release hydrocortisone in patients with adrenal insufficiency: a phase 3b, open-label, extension study. Eur J Endocrinol.

[39] Jones CM, Mallappa A, Reisch N, Nikolaou N, Krone N, Hughes BA, O’Neil DM, Whitaker MJ, Tomlinson JW, Storbeck KH, Merke DP, Ross RJ, Arlt W (2017). Modified-Release and Conventional Glucocorticoids and Diurnal Androgen Excretion in Congenital Adrenal Hyperplasia. J Clin Endocrinol Metab.

[40] Neumann U, Whitaker MJ, Wiegand S, Krude H, Porter J, Davies M, Digweed D, Voet B, Ross RJ, Blankenstein O (2018). Absorption and tolerability of taste-masked hydrocortisone granules in neonates, infants and children under 6 years of age with adrenal insufficiency. Clin Endocrinol (Oxf).

[41] Oksnes M, Björnsdottir S, Isaksson M, Methlie P, Carlsen S, Nilsen RM, Broman JE, Triebner K, Kämpe O, Hulting AL, Bensing S, Husebye ES, Løvås K (2014). Continuous subcutaneous hydrocortisone infusion versus oral hydrocortisone replacement for treatment of addison’s disease: a randomized clinical trial. J Clin Endocrinol Metab.

[42] Nella AA, Mallappa A, Perritt AF, Gounden V, Kumar P, Sinaii N, Daley LA, Ling A, Liu CY, Soldin SJ, Merke DP (2016). A Phase 2 Study of Continuous Subcutaneous Hydrocortisone Infusion in Adults With Congenital Adrenal Hyperplasia. J Clin Endocrinol Metab.

[43] Al Nofal A, Bancos I, Benkhadra K, Ospina NM, Javed A, Kapoor E, Muthusamy K, Brito JP, Turcu AF, Wang Z, Prokop L, Erickson DZ, Lteif AN, Natt N, Murad MH (2017). Glucocorticoid Replacement Regimens In Chronic Adrenal Insufficiency: A Systematic Review And Meta-Analysis. Endocr Pract.

[44] Auchus RJ, Buschur EO, Chang AY, Hammer GD, Ramm C, Madrigal D, Wang G, Gonzalez M, Xu XS, Smit JW, Jiao J, Yu MK (2014). Abiraterone acetate to lower androgens in women with classic 21-hydroxylase deficiency. J Clin Endocrinol Metab.

[45] Turcu AF, Spencer-Segal JL, Farber RH, Luo R, Grigoriadis DE, Ramm CA, Madrigal D, Muth T, O’Brien CF, Auchus RJ (2016). Single-Dose Study of a Corticotropin-Releasing Factor Receptor-1 Antagonist in Women With 21-Hydroxylase Deficiency. J Clin Endocrinol Metab.

